# Are YouTube™ and TikTok™ videos useful as educational tool for patients with cleft lip and palate?

**DOI:** 10.1590/2177-6709.29.6.e2424151.oar

**Published:** 2025-01-13

**Authors:** José Dilson Alves de OLIVEIRA, Anna Vitória Mendes Viana SILVA, Carina Cristina MONTALVANY-ANTONUCCI, Gabriele Andrade MAIA, Kamila Rodrigues Junqueira CARVALHO, Soraia MACARI

**Affiliations:** 1 Federal University of Minas Gerais, School of Dentistry, Department of Restorative Dentistry (Belo Horizonte/MG, Brazil).; 2 Federal University of Minas Gerais, School of Dentistry, Department of Child and Adolescent Oral Health (Belo Horizonte/MG, Brazil).

**Keywords:** Cleft lip, Cleft palate, Social media, Treatment outcome, Dental health education, Health care, Fissura labial, Fissura palatina, Mídias sociais, Resultado do tratamento, Educação em Saúde Bucal, Atenção à saúde

## Abstract

**Objective:**

To evaluate the quality of YouTube™ and TikTok™ videos as educational tools for patients with cleft lip and palate (CLP) as regards their care, and multidisciplinary treatment.

**Methods:**

Videos were searched on YouTube™ and TikTok™ using four keywords. The reliability and quality of the first 60 videos for each keyword and platform were analyzed. The following variables were analyzed: the source, distribution, and purpose of the videos, the general and audiovisual quality of the videos, and their main subject. The study’s covariates were cleft classification, dental treatment, pre-surgical orthopedic treatments, surgical and medical treatments.

**Results:**

Of the 480 videos selected, 303 videos were evaluated (177 excluded due to the exclusion criteria). TikTok™ emerged as the most frequently accessed platform, recording a greater number of views and likes. YouTube™ stood out for its availability of longer and more comprehensive videos, in terms of content. On YouTube™ the majority of videos were produced by academic/health and medical organizations, predominantly aimed at educational purposes; whereas on TikTok™ prevailed the production of individual and personal content geared toward informational purposes. On both platforms, the videos proved to be of low quality. YouTube™ videos from individual sources and organizations were associated with medium and low quality, respectively. Additionally, YouTube™ videos of shorter duration were of lower quality. TikTok™ videos had lower overall quality, especially those produced individually, regardless of associations.

**Conclusions:**

YouTube™ and TikTok™ exhibited predominantly low-quality videos, suggesting they are not suitable as educational tools to guide patients with CLP for their multidisciplinary treatment.

## RESUMO

**Objetivo:**

Avaliar a qualidade dos vídeos do YouTube™ e TikTok™ como ferramentas educacionais para pacientes com fissura labiopalatina (FLP), no que diz respeito aos cuidados e tratamento multidisciplinar.

**Métodos:**

Vídeos foram pesquisados no YouTube™ e TikTok™ utilizando quatro palavras-chave. A confiabilidade e qualidade dos primeiros 60 vídeos para cada palavra-chave e plataforma foram analisadas. As variáveis analisadas foram: fonte, distribuição e propósito dos vídeos, qualidade geral e audiovisual dos vídeos, e seu principal assunto. As covariáveis do estudo foram classificação da fissura, tratamento odontológico, tratamentos ortopédicos pré-cirúrgicos, tratamentos cirúrgicos e médicos.

**Resultados:**

Dos 480 vídeos selecionados, 303 foram avaliados (177 excluídos devido aos critérios de exclusão). O TikTok™ emergiu como a plataforma mais frequentemente acessada, registrando um maior número de visualizações e curtidas. O YouTube™ destacou-se pela disponibilidade de vídeos mais longos e mais abrangentes em termos de conteúdo. No YouTube™, a maioria dos vídeos foi produzida por organizações acadêmicas/saúde e médicas, predominantemente com fins educacionais, enquanto no TikTok™ prevaleceu a produção de conteúdo individual e pessoal, voltado para fins informativos. Em ambas as plataformas, os vídeos mostraram-se de baixa qualidade. Vídeos do YouTube™ de fontes individuais e organizações foram associados a qualidade média e baixa, respectivamente. Além disso, vídeos do YouTube™ de menor duração apresentaram qualidade inferior. Vídeos do TikTok™ tiveram qualidade geral inferior, especialmente aqueles produzidos individualmente, independentemente de associações.

**Conclusão:**

YouTube™ e TikTok™ exibiram predominantemente vídeos de baixa qualidade, sugerindo que não são adequados como ferramentas educacionais para orientar pacientes com FLP em seu tratamento multidisciplinar.

## INTRODUCTION

Cleft lip and palate is a congenital deformity that occurs due to lack of fusion between the maxillary segments and/or palate during embryogenesis, and affects approximately 1/650 live births worldwide,^1^ ^,^ ^2^ and 1/1,924 in Brazil.^3^ Individuals affected by clefts require multidisciplinary treatment, beginning at birth and possibly extending throughout life.^4^ ^,^ ^5^ The procedures cover a variety of interventions, from pre-surgical orthopedic treatments, such as nasoalveolar molding (NAM),^4^ ^,^ ^6^ and even corrective surgeries, including palatoplasty and cheiloplasty, as well as oral and maxillofacial procedures in adulthood.^5^ Furthermore, they may need dental and orthodontic treatments, speech therapy, and otorhinolaryngology, among others.^5^

In this scenario, many patients with cleft lip and palate, along with their families, face several uncertainties regarding the treatment stages, requiring accurate information about their condition.^7^ ^,^ ^8^ While it has traditionally been the responsibility of healthcare professionals and organizations to provide information about treatment, the ubiquity of Internet access means that the majority of the world’s population has a wide range of information sources available. The Internet has become the main reference not only for medical data, but for different areas of the health system,^9^ ^-^ ^16^ and also to search for diagnoses or treatments before even seeking a professional evaluation.^7, 17-19^ Despite the potential benefits of on-line health information, where everyone can share their opinions, the diversity of this content on the Internet ranges from professional information to patients’ personal experiences.^20^ ^,^ ^21^ This makes the possibility of spreading misinformation and erroneous content a concern,^18^ ^,^ ^19^ as patients may not have the necessary skills to evaluate medical information and associate it with their health status.^19^ ^,^ ^21^

Among the most widely used social networks on the Internet are YouTube™ and TikTok™.^17^ ^,^ ^22^ YouTube™ is the video-sharing platform of choice, and has become a phenomenon for commercial and personal content distribution, as well as for social media, due to its ability to provide visual and audio information. It is now the second most popular website in the world after Google, with more than a billion users.^17^ ^,^ ^22^ In addition to having diverse video content, YouTube™ also allows access to a wide variety of health-related videos.^19^ TikTok™, a popular social media video-sharing platform, was launched in 2016 and quickly rose to prominence in the market. In 2018 and 2019, it won the title of most downloaded application, with 800 million active users globally, registering billions of videos viewed daily.^17^ ^,^ ^22^ Due to its time restriction for videos, ranging from 15 seconds to 3 minutes, TikTok™ stands out as an active learning tool thanks to its short, condensed, and fun approach.^23^ However, the reliability, accuracy, and scientific validity of information, especially related to health, raise doubts, since videos uploaded to these platforms do not undergo peer review.^18^ ^,^ ^23^ Therefore, the present study aimed to evaluate and compare YouTube™ and TikTok™ videos as educational tools for patients with cleft lip and palate, in terms of reliability, quality, content, and specific features of the videos, concerning the etiology, their care, and multidisciplinary treatment.

## MATERIAL AND METHODS

### VIDEO SELECTION

It is worth noting that this study did not require approval from the ethics committee, as it contains public data. Two video-sharing sites, YouTube™ (www.youtube.com) and TikTok™ (www.tiktok.com), were used to conduct a search on January 10 and 11, 2023, respectively, for videos containing information for cleft lip and palate, using the following keywords: “cleft lip and palate and dentist”, “cleft lip and palate dental care plan”, “lip and palate and lip surgery” and “dental treatment in a patient with cleft lip and palate”, as they are the terms that generated the most results in both platforms. The videos were searched using combinations of the four keywords for each platform. More than 95% of people view only the first 60 videos in an online search.^20^ In this way, the first 60 results for each keyword on each platform were selected, using videos in English and Spanish as inclusion criteria, totaling 480 videos (240 on each platform).

The exclusion criteria were: information unrelated to cleft lip and palate, duplicate videos and videos containing advertising.^19^ As the dynamics and nature of the two platforms are different, the search filters are not standardized, and therefore the search histories and cookies were excluded in advance^18^, the platforms were accessed without logging in.

### VIDEO ANALYSIS

The information from the videos was evaluated using a checklist prepared by experts in cleft lip and palate, and filled out on Google Forms. For each video, information about its title, upload date, country of origin, and language was extracted, and the following general parameters were recorded: (1) number of views; (2) duration (minutes); (3) number of comments; and (4) total number of “likes” and “dislikes”, and (5) time since upload (years). The videos were also categorized according to their source, into four basic groups: (1) media (video presented by an identified news source/media), (2) individual (video presented by an individual), (3) academic/health organization (video presented by an academic conference, research group, or medical organization), and (4) consumer (video endorsing and/or promoting the sale of a product/service). The videos were distributed concerning the person who generated the information: (1) clinical dentist, (2) specialist dentist, (3) staff, (4) educators, (5) doctor, (6) charity/NGO, and (7) television programs.

The main objective of the videos was also investigated and categorized into four headings: (1) educate (the video informs/teaches about the topic, which includes evidence-based information), (2) opinion (video that portrays the perspective of an individual or organization on the subject), (3) academic presentation (video of a presentation for an academic audience, e.g. conference proceedings), and (4) commercial (video that promotes the product(s) of a company or individual). The objective classification was based on the subject that was predominantly in focus.

An analysis was also carried out regarding the content of the information provided in the videos: (1) etiology, (2) gestational period, (3) prevalence, (4) classification of the cleft (Lahshal, Spina, or Veau),^24^ ^,^ ^25^ (5) medical/surgical treatment, (6) dental treatment (prenatal diagnosis, breastfeeding, diet, oral and dentition changes, information on the use of nasoalveolar molding (NAM), nasal taper and elevators, and palate obturator prostheses), and (7) other matters. The videos were scored according to the veracity of each information present: (0) the video did not provide information regarding the veracity of its content, (1) incorrect content (Fake news), (2) content out of date, and (3) correct content. The videos were searched on YouTube™ and TikTok™ using combinations of the keywords, resulting in the 60 videos analyzed. The reliability and quality of these videos were assessed. The variables analyzed included the source, distribution, and purpose of the videos, general and audiovisual quality, and the main subject. The covariates of the study were the classification of the cleft, dental treatment, pre-surgical orthopedic treatments, and surgical and medical treatments.

The audiovisual quality of the videos followed the criteria according to Sorensen et al.^26^ Videos that included clear visuals, text, professional graphics, or effects, and that caused no difficulty in understanding spoken words and music were rated as good. Home videos, videos with fair quality and average text clarity, difficult-to-understand speech, and distracting audio or sounds in the background were rated as moderate. Videos that were blurry, grainy, with hard-to-understand visuals, and/or had no audio were rated as poor. The content quality assessment was carried out according to Hegarty et al.^27^ Videos with excellent quality and flow, containing most of the relevant information and that were very useful to patients were classified as excellent. Videos with moderate quality and suboptimal flow, in which some important information was discussed adequately, but others were poorly discussed, and that were somewhat helpful to patients, were classified as moderate. Videos with poor quality and flow, in which some information was listed but mostly missing, as well as videos that were of no use to the patients, were rated as poor. Interaction index formulas were calculated for each video to assess the level of interaction, using the number of likes, dislikes, and total views, according to Hassona et al.^28^

To measure the reliability of the videos, an inter- and intra-rater calibration was performed on the first ten videos from both platforms (YouTube™ and TikTok™). Two experienced pediatric dentists (A.V.M.V. and K.R.J.C.) were selected for the evaluation. Initially, a detailed checklist was developed by experts in cleft lip and palate, containing relevant topics such as etiology, gestational period, prevalence, cleft classification, medical/surgical treatment, dental treatment, and other topics. The raters then participated in an initial training session, to ensure a uniform understanding of the evaluation criteria. The first ten videos from each platform were evaluated independently by both raters using the developed checklist. The Kappa coefficient was calculated to determine the degree of agreement between the two raters, resulting in a value of 0.99, indicating excellent agreement. After a two-week interval, the same ten videos were re-evaluated by the raters to check the consistency of the ratings over time, and again, high Kappa values were obtained, confirming the consistency of the individual ratings. Following the test and retest ratings, the raters discussed any remaining discrepancies and adjusted the checklist criteria, to ensure consistent and reliable ratings of all subsequent videos. These steps ensured that the video ratings were performed consistently and reliably, ensuring the validity of the study results.

## STATISTICAL ANALYSIS

The Kappa coefficient was used to assess agreement between reviewers. Data organization and statistical analysis were performed using Microsoft Excel (Microsoft) and the Statistical Package for Social Sciences (SPSS for Windows, version 21.0, IBM Inc., Armonk, NY, USA). To characterize the video resources, the frequency distribution of the data was determined, and the percentages for the categorical variables were calculated. One-way analysis of variance and Kruskal-Wallis tests were used to compare video parameters among videos of good, moderate, and poor informative content. The chi-square test of the linear trend was used to determine the platform outcome (Tiktok™ or YouTube™) and the polytomous nominal qualitative variable. Statistical significance was set at p < 0.05.

## RESULTS

**Figure 1:**
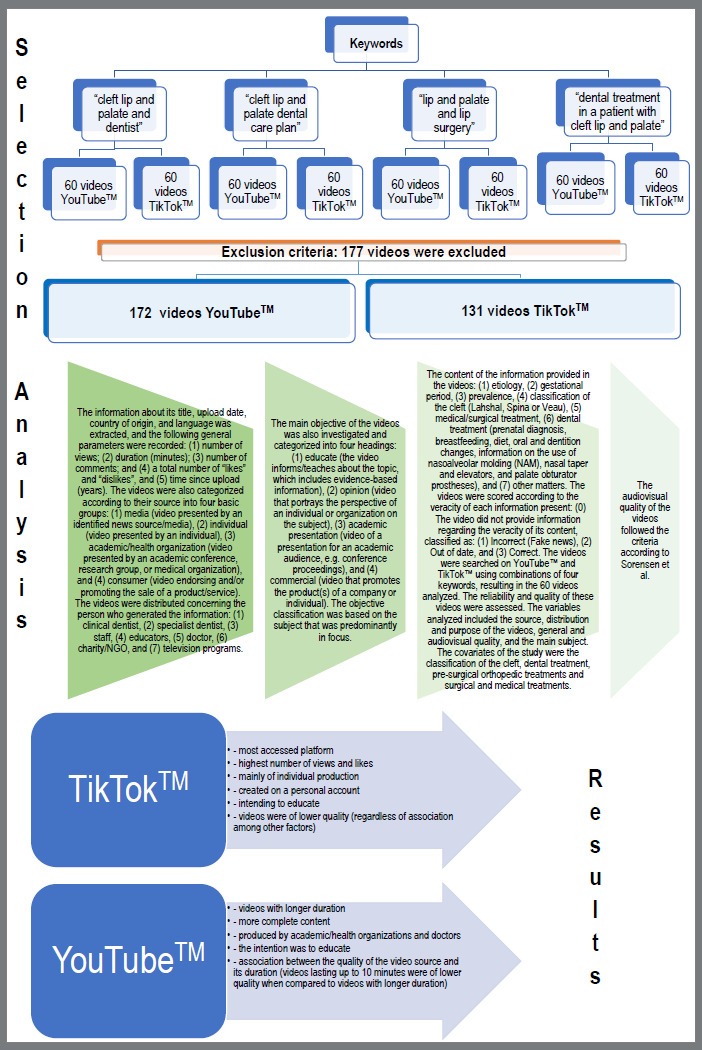
The schematic chart summarizes the video selection, analysis, and main findings.

Of the 480 videos selected (4 keywords, the first 60 videos, and 2 platforms), 177 were excluded due to the exclusion criteria. In total, 303 videos were evaluated, 172 from the YouTube™ platform and 131 from TikTok™. For YouTube™ videos, variables such as the number of views, likes, and dislikes; the number of comments; and the number of years since the videos were uploaded, were evaluated (Fig 1). The same variables were evaluated for TikTok™, except for dislikes (Table 1). Furthermore, for TikTok™ videos, the number of saves that the video received was evaluated (Table 1). TikTok™ videos, although they had been uploaded for fewer years, had a higher number of views, likes, and comments, when compared to YouTube™ (Table 1).

**Table 1:** Descriptive analysis of the investigated data (n = 303).

Platform	YouTube™					TikTok™					
Variables	Minimum	Maximum	Average	Median	Standard deviation	Minimum	Maximum	Average	Median	Standard deviation	P value
Views	33	1,297,646.0	34,216.7	3,612.5	119,174.041	100	50,600,000.0	786,980.95	6,529	5,264,481.142	0.001
Likes	0	7,303.0	252.99	29.5	746,909.0	0	1,400,000.0	31,925.0	952	153,493.145	0.000
Dislikes	0	9.0	0.16	0.0	1.2	0	14.0	443.43	25	2,114.914	0.897
Comments	0	38.9	17.19	2.0	38,936.0	0	2,598.0	30.16	0	249,795.0	0.000
Years since upload	1	20.0	4.42	3.0	3.65	0	4.0	1.06	1.00	1.03	0.000
Saved	-	-	-	-	-	0	2,598.0	30.16	0	7,487.0	-

Kolmogorov-Smirnov test, Mann-Whitney test.

**Figure 2:**
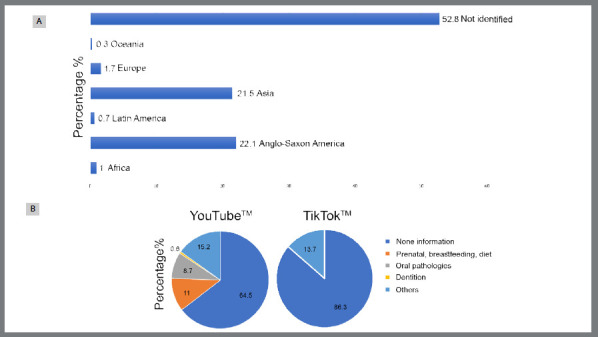
A) Data in percentage (%) of the continent of origin of TikTok™ and YouTube™ videos on cleft lip and palate (n = 303). B) Percentage (%) of subjects about dental treatment in TikTok™ and YouTube™ videos.

Videos in English and Spanish were evaluated, of which 284 videos (93.7%) were in English and only 19 (6.3%) were in Spanish. Figure 2A illustrates the continent of origin of TikTok™ and YouTube™ videos, but in 52.8% of the videos it was not possible to identify the origin. Most of the videos posted on the topic came from Anglo-Saxon America (22.1%) and Asia (21.5%). Regarding the country of origin, in most videos, it was not possible to identify the country, but the highest frequency of publications was from the United States (20.8%), India (17.8%), and Saudi Arabia (0.9%) (data not shown).

The source of the videos, their distribution, and their objectives differed statistically between the studied platforms (Table 2). On YouTube™, there was a predominance of videos made by organizations, carried out by doctors, and with an educational goal (Table 2). On TikTok™, the prevalence was of videos of personal stories, made by laypeople providing personal information (Table 2).

**Table 2:** Variables evaluated in the videos (n = 172 YouTube™ / n = 131 TikTok™) and main themes treated in the YouTube™ (n = 172) and TikTok™ videos (n = 131).

Platform	YouTube™	TikTok™	
Variable	n (%)	n (%)	P-value
Video source			
Media	5 (2.9)	2 (1.5)	0.000
Individual users	61 (35.5)	126 (96.2)	
Organization	94 (54.7)	2 (2.3)	
Consumer	12 (7.0)	0 (0.0)	
Video distribution			
Clinical dentist	13 (7.6)	8 (6.1)	0.000
Specialist dentist	45 (26.2)	27 (20.6)	
Individual users	21 (12.2)	77 (58.8)	
Educators	12 (7.0)	2 (1.5)	
Doctor	72 (41.9)	14 (11.7)	
Charity/non-governmental organizations (NGOs)	9 (5.2)	3 (2,3)	
TV program	0 (0.0)	0 (0.0)	
Purpose of the video			
To educate	76 (44.2)	51 (38.9)	0.001
Opinion	51 (29.7)	78 (59.5)	
Academic presentation	13 (7.6)	0 (0.0)	
Commercial	32 (18.6)	2 (1.5)	
Main theme			
Etiology	50 (29.1)	3 (2.3)	0.000
Gestational period when the cleft lip occurred	4 (2.3)	3 (2.3)	
Prevalence	0 (0.0)	3 (2.3)	
Medical treatment	47 (27.3)	29 (22.1)	
Dental treatment	24 (14.0)	28 (21.4)	
Other matters	43 (25.0)	58 (44.3)	

Chi-square test for trend.

The main themes covered in the videos were significantly different when the platforms were compared to each other (Table 2). The themes of etiology, gestational period in which the cleft occurs, and medical treatment were more prevalent in YouTube™ videos when compared to TikTok™ (Table 2). The three main topics most covered in descending order on YouTube™ were: etiology, medical treatment, and other subjects (personal stories); while on TikTok™, other subjects (personal stories) were among the most discussed topics, followed by medical and dental treatment (Table 2).

Figure 2B demonstrates that among the main topics related to dental treatment in individuals with cleft lip and palate, it was found that only 35.5% of YouTube™ videos presented scientifically based topics on prenatal diagnosis, breastfeeding, diet, oral changes, and dentition. On TikTok™, many of the videos did not present information or there was a predominance of videos with individuals reporting their personal experiences (Fig 2B).

Information from videos on both platforms was categorized as non-existent, correct, incorrect, and outdated (Table 3). Except for the content of the videos containing information on the use of NAM, nasal taper, nasal elevator, and palate obturator prostheses, where there was no statistical difference between YouTube™ and TikTok™; other subjects -such as cleft classification, general information, dental treatment in individuals with clefts, and surgical medical treatment- differed statistically from each other; with YouTube™ presenting videos with more correct information when compared to TikTok™ (Table 3). YouTube™ showed no videos with incorrect information, while TikTok™ showed 1.5% erroneous information regarding general information. YouTube™ had a greater amount of outdated content when compared to TikTok™ videos (Table 3). The vast majority of videos did not provide information about cleft classification; however, 14 YouTube™ videos and one TikTok™ video addressed cleft classification; however, only 8.2% of the videos cited some type of diagnostic criteria, namely Lahshal (2.9%), Spina (4.1%), and Veau (1.2%).

**Table 3:** Information provided in YouTube™ (n = 172) and TikTok™ (n = 131) videos, and general and audiovisual quality of videos concerning cleft lip and palate in YouTube™ (n = 172) and TikTok™ (n = 131) videos.

Platform	YouTube™	TikTok™	P-value
Type of information	n (%)	n (%)	
Cleft classification			
The video does not provide the information	158 (91.9)	130 (99.2)	0.003
Incorrect (Fake News)	0 (0.0)	0 (0.0)	
Correct	14 (8.1)	1 (0.8)	
Outdated	0 (0.0)	0 (0.0)	
General information			
The video does not provide the information	94 (54.7)	122 (93.1)	0.000
Incorrect (Fake News)	0 (0.0)	2 (1.5)	
Correct	74 (43.0)	7 (5.3)	
Outdated	4 (2.3)	0 (0.0)	
Dental treatment in individuals with cleft			
The video does not provide the information	133 (77.3)	123 (93.9)	0.000
Incorrect (Fake News)	0 (0.0)	0 (0.0)	
Correct	35 (20.3)	8 (6.1)	
Outdated	4 (2.3)	0 (0.0)	
Use of NAM plates, taper, nasal elevator and palate obturator prosthesis			
The video does not provide the information	142 (82.5)	105 (80.2)	0.532
Incorrect (Fake News)	0 (0.0)	0 (0.0)	
Correct	28 (16.3)	26 (19.8)	
Outdated	2 (1.2)	0 (0.0)	
Surgical medical treatment			
The video does not provide the information	121 (70.3)	118 (90.1)	0.000
Incorrect (Fake News)	0 (0.0)	0 (0.0)	
Correct	48 (28.0)	11(8.4)	
Outdated	3 (1.7)	2 (1.4)	
Overall quality			
High	44 (25.6)	2 (1.5)	0.000
Average	57 (33.1)	34 (26.0)	
Low	71 (41.3)	95 (72.5)	
Audiovisual quality			
Good	41 (23.8)	10 (7.6)	0.241
Moderate	73 (42.4)	86 (65.6)	
Bad	58 (33.7)	35 (26.7)	

Chi-square test for trend.

On both platforms, the general quality of the videos was classified as low, although the audiovisual quality was moderate (Table 3). Only the general quality of the videos differed statistically between YouTube™ and TikTok™, the latter having lower quality videos (Table 3).

Bivariate analyses were carried out to evaluate the association between the general quality of the video and independent variables, such as the source, distribution, and objective of the video (Tables 6 and 7). Only on YouTube™ the video source was associated with quality, in which videos from individual sources presented medium quality and videos from organizations were mostly of low quality ( Table 4). On TikTok™, there were no significant associations, although it was possible to observe that individually produced videos were of low quality (73%), as were personally distributed videos (77.9%) (Table 4).

**Table 4:** Association between quality of YouTube™ videos and information from videos about cleft lip and palate, and association between quality of TikTok™ videos and information from videos about cleft lip and palate.

YouTube™			
Variable	High	Medium	Low
Video Source** n (%)			
Media	2 (28.6)	2 (28.6)	3 (42.9)
Individual users	20 (10.7)	52 (27.8)	115 (61.5)
Organization	21 (21.6)	33 (34.0)	43 (44.3)
Consumers	3 (25.0)	4 (33.3)	5 (41.7)
Video distribution** n (%)			
Clinical dentist	0 (0.0)	2 (25.0)	6 (75.0)
Specialist dentist	1 (3.7)	10 (37.0)	16 (59.3)
Individual users	1 (1.3)	16 (20.8)	60 (77.9)
Educators	0 (0.0)	1 (50.0)	1 (50.0)
Doctors	0 (0.0)	5 (35.7)	9 (64.3)
Charity/non-governmental organizations (NGOs)	0 (0.0)	0 (0.0)	3 (100)
Television program	0 (0.0)	0 (0.0)	0 (0.0)
Purpose of the video** n (%)			
Educate	20 (15.7)	47 (37.0)	60 (47.2)
Opinion	16 (12.4)	33 (25.6)	80 (62.0)
Academic presentation	3 (23.1)	4 (30.8)	6 (46.2)
Commercial	7 (20.6)	7 (20.6)	20 (58.8)
TikTok™			
Variable	High	Medium	Low
Video Source** n (%)			
Media	0 (0.0)	1 (50.0)	1 (50.0)
Individual	2 (1.6)	32 (25.4)	92 (73.0)
Organization	0 (0.0)	1 (33.3)	2 (66.7)
Consumer	0 (0.0)	0 (0.0)	0 (0.0)
Video distribution* n (%)			
Clinical dentist	0 (0.0)	2 (25.0)	6 (75.0)
Specialist dentist	1 (3.7)	10 (37.0)	16 (59.3)
Individual users	1 (1.3)	16 (20.8)	60 (77.9)
Educators	0 (0.0)	1 (50.0)	1 (50.0)
Doctors	0 (0.0)	5 (35.7)	9 (64.3)
Charity/non-governmental organizations (NGOs)	0 (0.0)	0 (0.0)	3 (100)
Television program	0 (0.0)	0 (0.0)	0 (0.0)
Purpose of the video** n (%)			
Educate	1 (2.0)	17 (33.3)	33 (64.7)
Opinion	1 (1.3)	17 (21.8)	60 (76.9)
Academic presentation	0 (0.0)	0 (0.0)	0 (0.0)
Commercial	0 (0.0)	0 (0.0)	2 (100.0)

*Pearson chi-square test. ** Chi-square test for trend. ***Fisher exact test.

Table 5 demonstrates the associations between the general quality of the video with the subject covered and the duration time, the latter being categorized as up to 10 minutes and over 10 minutes for YouTube™, and up to 1 minute and over 1 minute for TikTok™. Only the subject of etiology was associated with high quality, while prevalence, medical and dental treatment, and individual reports were associated with low quality, the latter having the highest incidence among videos from both platforms (Table 5). The duration of the videos varied greatly, depending on the platform, with shorter duration videos being found in TikTok™ than in YouTube™ videos (Table 5). The duration time was categorized, and it was observed that on TikTok™ 75.6% of the videos were less than 30 seconds long, 22.1% were between more than 30 seconds and less than 2 minutes, and only 2.3% of the videos were more than 2 minutes long (data not shown). YouTube™ videos were longer and more complete in terms of information, in which 38.4% of the videos were less than 2 minutes long, 29.1% were between 2 and 5 minutes long, 11.6% were between 6 and 10 minutes, 13.4% lasted between 11 and 20 minutes, and 11% lasted more than 40 minutes, with a duration extending to more than an hour (data not shown). Although there was a divergence in the length of videos between the platforms, and the fact that 73.4% of TikTok™ videos showed low quality in videos up to 1 minute, only on YouTube™ there was an association between video duration and quality, with 50% of the videos of up to 10 minutes being of low quality (Table 5).

**Table 5:** Association between overall quality of the videos and the theme and time duration of the video.

Variable	High	Medium	Low	P-value
Theme of the video** n (%)				
Etiology	27 (50.9)	17 (32.1)	9 (17.0)	0.00
Gestational period	0 (0.0)	4 (57.1)	3 (42.9)	
Prevalence	0 (0.0)	0 (0.0)	3 (100)	
Classification of cleft	5 (45.5)	5 (45.5)	1 (9.1)	
Medical treatment	11(14.5)	28 (36.8)	37 (48.7)	
Dental treatment	4 (7.7)	21 (40.4)	27 (51.9)	
Other (ex. self-report)	3 (3.0)	16 (15.8)	82 (81.2)	
Time duration YouTube™* n (%)				
Up to 10 minutes	24 (17.6)	44 (32.4)	68 (50.0)	0.00
More than 10 minutes	20 (55.6)	13 (36.1)	3 (8.3)	
Time duration TikTok™* n (%)				
Up to 2 minute	2 (1.6)	32 (25.0)	94 (73.4)	0.17
More than 2 minute	0 (0.0)	2 (66.7)	1 (33.3)	

*Pearson chi-square test **Chi-square test for trend ***Fisher exact test.

## DISCUSSION

This study aimed to evaluate the content of YouTube™ and TikTok™ videos in the treatment of individuals with cleft lip and palate. The study revealed that TikTok™ was the most accessed platform, with the highest number of views and likes. On the other hand, YouTube™ presented videos with longer duration and more complete content. On YouTube™, the majority of videos were produced by academic/health organizations and doctors, with the intent to educate. In contrast, on TikTok™, the videos were mainly of individual production, created on personal accounts, also intending to educate. YouTube™ videos showed an association between the quality of the video source and its duration, videos lasting up to 10 minutes were of lower quality when compared to videos with longer duration, while TikTok™ videos were of lower quality, regardless of association among other factors.

The role of social media in health is complex and multifaceted, covering different areas of health and knowledge. It may have advantages, obstacles, and the necessity for quality oversight. Social networks are freely accessible to the public, enabling a diverse range of individuals -including nonspecialists, health professionals, and researchers- to share personal experiences and scientifically-based content, respectively. Social media is present in various aspects of global health including improving healthcare disaster decision-making during the COVID-19 pandemic, cardiovascular healthcare, pregnancy care, nutrition, and obesity. There can be benefits to discussing healthcare on social media, and the healthcare professionals need to be engaged with social media.^9^ ^-^ ^16^ In this way, the current research sought to assess and compare YouTube™ and TikTok™ videos as educational resources for patients with cleft lip and palate, focusing on their reliability, quality, content, and particular attributes related to the cause, management, and interdisciplinary treatment.

This study showed that TikTok™ stood out as the most frequently accessed platform, accumulating the highest number of views and likes, although a lower quality of content was perceived. These findings are in line with the conclusions of Meade and Dreyer,^23^ who identified low-quality content and reliability in videos on TikTok™ related to orthodontic retention. On the other hand, YouTube™ stood out for presenting longer and more comprehensive videos in terms of content. Contrary to the results of this study, Korkmaz and Buyuk^19^ analyses of videos on YouTube™ related to cleft lip and palate treatment revealed that the majority were classified as a moderate quality in terms of information, although they were not considered an entirely reliable source for patients.

Both platforms, characterized by their dynamicity, reflected constantly evolving research results, shaped by continuous changes in audience interests and viewing patterns. It is important to highlight that variables such as viewing rate, likes, and dislikes may be susceptible to manipulation. Although the keywords were selected based on Google Trends, aiming to identify the most used terms, it is important to bear in mind that different sets of keywords can lead to the discovery of different videos.^19^

The videos on both platforms were predominantly in English, with no specific indication of their geographic origin. Consistent with the present results, previous studies found that patient education materials provided by cleft lip and palate teams, as well as personal videos shared on online platforms, were more available in English than in Spanish.^7^ ^,^ ^8^

On YouTube™, the videos were mostly produced by academic/health organizations and doctors, with the aim of educating; whereas on TikTok™, the video production was individual and personal, intending to give an opinion. Ideally, videos with greater educational content should be more suitable for the patient; however, it was found that on YouTube™, individual videos of lower quality had greater potential for user engagement.^20^ The patient often lacks the intrinsic ability to evaluate the quality of the video. At the same time, the quality of the content does not always extend to medical videos from reputable entities created for educational purposes.^20^ The use of social media is one of the most common activities on the Internet, and has become more widespread with the increased use of mobile devices, providing a new dimension through which to access and provide health-related information for both doctors and patients.^18^ In this sense, health professionals must be cautious when directing their patients to the appropriate use of YouTube™ and TikTok™ videos.^19^ ^,^ ^28^ ^,^ ^29^

In the study conducted by Korkmaz and Buyuk,^19^ a notable interest in videos related to cleft lip and palate treatment was found among users. The number of views of these videos reached significant levels, and the public regularly expressed their engagement through comments, sharing information, and personal experiences. In this study, when comparing videos on cleft lip and palate treatment on both platforms, it was observed that YouTube™ videos were more extensive and comprehensive in terms of information, containing topics related to breastfeeding, prenatal care, diet, changes in oral health, and dentitions. By contrast, on TikTok™, we found no videos that addressed the topic in depth. However, it is important to highlight that this type of subjective opinion present on social media can lead to inherent risks for the patient’s health.^20^ The presence of incorrect information can compromise patients’ decision-making, discouraging the search for appropriate treatment or wrongly directing them to care alternatives that are not supported by science.^20^

Regarding the quality of the videos found, it was observed that the majority of TikTok™ videos were of significantly low quality. Although a clear association between the information contained in cleft lip and palate videos and the overall quality of TikTok™ videos was not identified, those containing personal opinions consistently demonstrated lower quality, when compared to videos for educational, academic, and commercial purposes. In the study conducted by Meade and Dreyer,^23^ which evaluated the reliability and quality of TikTok™ videos concerning orthodontic retention, it was found that the majority of videos were of low content and produced by laypeople, indicating that videos originating from this platform had little useful educational value for the viewer.

In the current study, in YouTube™ videos, quality was significantly associated with the video source and its duration. In contrast to the findings of this study, Hassona et al.^28^ analyzed videos on YouTube™ as a source of information regarding oral cancer, and found no significant association between the usefulness of the video, the viewing rate, viewer interaction, and video duration.

Regarding the platform, the current study corroborates with findings from Kilinç,^18^ which also found that videos on TikTok™ displayed lower quality and less reliable information, when compared to videos on YouTube™. Although many videos on TikTok™ are newer than those on YouTube™, they have demonstrated greater audience appeal. Notably, while similar numbers on YouTube™ were expressed in tens, on TikTok™, they reached hundreds of thousands. This can be interpreted as a rapid and widespread dissemination of misinformation to the public, which can be critical for public health.^30^ ^,^ ^31^

Videos are classified on platforms based on audience and interaction assessments. Videos of medium to long duration, posted outside business hours and during business days, as well as those that use subjective language, associated with less popular events, and including temporal indications, are more likely to attract views, likes, and comments. Furthermore, the incorporation of negative or low-arousal emotions emerges as an effective strategy to arouse general interest in a video on the platform.^32^ In this way, useful videos may not be ranked first in the viewing list and, therefore, may not be watched by viewers.^28^

An inherent limitation of this study is the lack of validated tools in the literature, given their innovative nature. This made it impossible to perform a multinomial regression analysis. Furthermore, the number of videos evaluated and the inconclusive results due to lack of content may be a limitation of this study. However, bivariate analyses have already presented important results regarding the provision of information and its quality regarding the topic, raising a warning about reliable search sources. This reinforces the need for control by health professionals, institutions, and organizations that provide assistance and scientific information when promoting quality content, sending useful videos, and guiding patients to reliable sources of information, in addition to all oral and written guidance and information given to patients and guardians.^8^ It also confirms the need for more studies on the topic, to standardize analyses to investigate the quality of knowledge on the treatment of cleft lip and palate on different social platforms, as well as the development of guidelines to guide the most necessary and useful information for everyone. 

Social media platforms must provide clear, accessible, evidence-based public health information.^33^ YouTube™ and TikTok™ offer significant opportunities for disseminating health information, creating new data sources, and enhancing public health literacy.^9^ In this context, social media sites are key institutions shaping modern life, making it essential to provide health-promoting policies, programs, and information to optimize public health.^34^ Although there is evidence that indicates discordance in patients’ and professionals’ motives and use of social media in health care,^35^ as both social media present in this study continue to grow as platforms for health information, it is essential to prioritize accuracy and reliability to enhance patients’ self-care abilities and promote public health.^36^ Cleft lip and palate are one of the most prevalent craniofacial anomalies worldwide, affecting about 1 in every 650 live births globally^2^ and 1 in every 1924 live births in Brazil,^3^ patients must have accurate access to information on social media.

Social media has enormous potential in teaching-learning processes, but one cannot expect immediate social changes.^37^ Social media platforms, through the dissemination of content in video format, demonstrate a remarkable ability to improve epidemiological disease surveillance, enable large-scale communication, promote health education, facilitate knowledge translation, and foster collaboration between health professionals,^38^ primarily in low and middle-income countries.^39^ The spread of misinformation or inadequately communicated information can contribute to the adoption of harmful health behaviors and trigger adverse health outcomes among consumers, as well as potentially incite hysteria and chaos.^20^ ^,^ ^39^ Organizations that employ social media platforms must prioritize the provision of information that is accurate, of adequate duration, and easy to understand.^40^ The active promotion of credible social media sites by government entities, healthcare professionals, and researchers, combined with educational initiatives on the appropriate use of social media, can significantly contribute to mitigating the harmful effects of misinformation.^40^ ^,^ ^41^ On the other hand, as verified in the present study, several elements may be present in the videos of these platforms that tend to harm the implementation of learning experiences, such as: the lack of reliable content, and high-quality videos, among others, including the presence of elements that lead to destruction, such as advertisements and warnings; the lack of a filter system; the search for and organization of information; mechanisms that attest to the veracity of information, such as mandatory literary references; or the lack of tools that have been validated in prior literature.^31^

## CONCLUSION

YouTube™ and TikTok™ exhibited predominantly low-quality videos, which might suggest they are not suitable as educational tools to guide patients with cleft lip and palate in their multidisciplinary treatment. Healthcare practitioners, educational institutions, and professional associations must bear the duty of enhancing the quality of the content of YouTube™ and TikTok™ videos regarding the treatment of patients with cleft. The creation and dissemination of high-quality educational content by uploading valuable and, ideally, peer-reviewed videos is crucial to enhance evidence-based public health, while also guiding patients towards trustworthy sources of information.
